# Supplementary material for: Who is meeting the strengthening physical activity guidelines by definition: A cross-sectional study of 253 423 English adults?
Source: PLoS One. 2022 May 4;17(5):e0267277. doi: 10.1371/journal.pone.0267277 (PMC9067886; doi:10.1371/journal.pone.0267277)
Supplement: S1 Table — (DOCX) [file pone.0267277.s001.docx]

**S1 Table – Activities included within each definition of strengthening activity.**

|  | **HSE** | **Evidence** | **Guideline** |
| --- | --- | --- | --- |
| **Most Frequently Reported Activities*** | | | |
| Running *^a^* | **🗸** | **🗸** |  |
| Cycling | **🗸** |  |  |
| Swimming *^b^* | **🗸** |  |  |
| Aerobics *^c^* | **🗸** |  |  |
| Weight training ***^1^*** | **🗸** | **🗸** | **🗸** |
| Circuit training ***^2^*** | **🗸** | **🗸** | **🗸** |
| Bodyweight exercise *^3^* | **🗸** | **🗸** | **🗸** |
| Football | **🗸** | **🗸** |  |
| Dance (classes) *^d^* | **🗸** |  |  |
| Free weights | **🗸** | **🗸** |  |
| Yoga | **🗸** | **🗸** | **🗸** |
| Golf | **🗸** |  |  |
| Hill-walking *^e^* | **🗸** |  |  |
| Pilates | **🗸** |  |  |
| Weightlifting ^4^ | **🗸** | **🗸** | **🗸** |
| Rowing | **🗸** |  |  |
| Tennis | **🗸** | **🗸** |  |
| Badminton | **🗸** |  |  |
| **Less Frequently Reported Activities**** | | | |
| *Equestrian* ^f^ | **🗸** |  |  |
| *Gymnastics* ^g^ | **🗸** | **🗸** |  |
| *Squash* | **🗸** |  |  |
| *Water-based class* | **🗸** |  |  |
| *Netball* | **🗸** |  |  |
| *Boxing ^i^* | **🗸** |  |  |
| *Cricket* | **🗸** |  |  |
| *Rugby Union* | **🗸** |  |  |
| *Basketball* | **🗸** |  |  |
| *Martial Arts ^j^* | **🗸** |  |  |
| *Hockey* | **🗸** |  |  |
| *Surfing* | **🗸** |  |  |
| *Rugby League* | **🗸** |  |  |
| *Volleyball* | **🗸** |  |  |
| *Ten-pin bowling* | **🗸** |  |  |
| *Water-skiing* | **🗸** |  |  |

**Legend Table S1.** Activities are reported in descending order of overall contribution 2 population-level physical activity EG running cycling and swimming were most often reported and Pattinson contributed the least 2 overall physical activity energy expenditure. Less frequently reported activities are shown in the lower part of the table. all of the activities listed contributed less than 1% to total physical activity energy expenditure

^a-^Includes athletics ^b^-includes diving, water-polo; ^c^-includes aerobics, step-aerobics and cardio-vascular fitness classes ^d^-includes artistic dance, dance instruction dance-based exercise classes eg Zumba ^e^- includes, rambling, hill-walking and mountain walking^. f^-includes all equestrian sports (horse riding, show jumping, dressage, cross-country hashing. ^g^-includes all gymnastics, trampolining cheerleading; ^i^-includes boxing fitness classes. ^J^-includes all martial arts judo, karate, ju-jitsu, taekwondo, Aikido, kickboxing and capoeira.

*^1^*-Includes ‘weights machines’ and ‘weights classes’ *^2^*-Includes Circuit training, Circuit training classes *^3^*-Includes bodyweight exercises, Bootcamp/military fitness *^4^*-Includes weightlifting and powerlifting
