# Supplementary material for: Who is meeting the strengthening physical activity guidelines by definition: A cross-sectional study of 253 423 English adults?
Source: PLoS One. 2022 May 4;17(5):e0267277. doi: 10.1371/journal.pone.0267277 (PMC9067886; doi:10.1371/journal.pone.0267277)
Supplement: S2 Table — (DOCX) [file pone.0267277.s002.docx]

**S2 Table. Percentage of males and females (19-65 years) meeting three definitions of the aerobic and strengthening activity guidelines according to age, deprivation, level of education, and disability status.**

| **Males** | **HSE** | |  | **Evidence** | |  | **Guideline** | |
| --- | --- | --- | --- | --- | --- | --- | --- | --- |
|  | **%** | **(se)** |  | **%** | **(se)** |  | ***%*** | **(se)** |
| **Age Group** |  |  |  |  |  |  |  |  |
| **19-34 years** | 34.8 | (0.5) |  | 24.3 | (0.4) |  | *9.3* | (0.4) |
| **35-49 years** | 28.6 | (0.3) |  | 18.8 | (0.3) |  | *6.9* | (0.2) |
| **50-64 years** | 20.6 | (0.3) |  | 11.9 | (0.2) |  | *4.7* | (0.2) |
| **Area level deprivation (IMD Quintile)** |  |  |  |  |  |  |  |  |
| **Q1 (Least)** | 33.3 | (0.5) |  | 21.7 | (0.4) |  | *7.7* | (0.4) |
| **Q2** | 30.6 | (0.5) |  | 20.3 | (0.4) |  | *7.7* | (0.4) |
| **Q3** | 29.8 | (0.7) |  | 20.3 | (0.6) |  | *7.7* | (0.5) |
| **Q4** | 27.4 | (0.5) |  | 17.7 | (0.4) |  | *7.2* | (0.4) |
| **Q5 (Most)** | 24.0 | (0.4) |  | 15.6 | (0.3) |  | *4.6* | (0.3) |
| **Highest Qualification Achieved** |  |  |  |  |  |  |  |  |
| **≥ Level 4** | 35.0 | (0.3) |  | 22.8 | (0.3) |  | *7.8* | (0.2) |
| **= Level 3** | 28.7 | (0.5) |  | 19.0 | (0.5) |  | *8.1* | (0.4) |
| **=Level 2** | 21.0 | (0.5) |  | 14.3 | (0.4) |  | *6.7* | (0.4) |
| **≤Level 1** | 16.0 | (0.7) |  | 10.3 | (0.9) |  | *4.5* | (0.6) |
| **Disability** |  |  |  |  |  |  |  |  |
| **No Disability** | 30.7 | (0.3) |  | 20.4 | (0.2) |  | *7.5* | (0.2) |
| **Non-limiting  Disability** | 26.0 | (0.6) |  | 16.2 | (0.5) |  | *7.0* | (0.4) |
| **Limiting  Disability** | 14.8 | (0.5) |  | 8.7 | (0.4) |  | *4.9* | (0.3) |
|  | **HSE** | |  | **Evidence** | |  | **Guideline** | |
| **Females** | **%** | **(se)** |  | **%** | **(se)** |  | ***%*** | **(se)** |
| **Age group** |  |  |  |  |  |  |  |  |
| **19-34** | 28.2 | (0.3) |  | 15.0 | (0.3) |  | *5.3* | (0.2) |
| **35-49** | 25.2 | (0.3) |  | 12.7 | (0.2) |  | *3.7* | (0.2) |
| **50-64** | 19.2 | (0.2) |  | 7.0 | (0.2) |  | *2.6* | (0.1) |
| **Area level deprivation (IMD Quintile)** |  |  |  |  |  |  |  |  |
| **Q1 (Least)** | 30.1 | (0.4) |  | 14.5 | (0.3) |  | *5.9* | (0.3) |
| **Q2** | 27.6 | (0.4) |  | 13.3 | (0.3) |  | *4.7* | (0.3) |
| **Q3** | 25.9 | (0.5) |  | 12.7 | (0.4) |  | *4.2* | (0.4) |
| **Q4** | 24.7 | (0.4) |  | 11.9 | (0.3) |  | *3.9* | (0.2) |
| **Q5 (Most)** | 19.3 | (0.3) |  | 9.0 | (0.2) |  | *3.2* | (0.2) |
| **Highest Qualification Achieved** |  |  |  |  |  |  |  |  |
| **≥ Level 4** | 30.8 | (0.2) |  | 15.6 | (0.2) |  | *4.8* | (0.2) |
| **= Level 3** | 24.4 | (0.4) |  | 11.5 | (0.3) |  | *4.3* | (0.3) |
| **=Level 2** | 18.2 | (0.3) |  | 7.6 | (0.2) |  | *3.2* | (0.2) |
| **≤ Level 1** | 12.0 | (0.8) |  | 4.9 | (0.5) |  | *3.1* | (0.5) |
| **Disability** |  |  |  |  |  |  |  |  |
| **No Disability** | 27.0 | (0.2) |  | 13.4 | (0.2) |  | *4.2* | (0.1) |
| **Non-limiting Disability** | 25.4 | (0.5) |  | 11.7 | (0.3) |  | *4.6* | (0.3) |
| **Limiting Disability** | 13.0 | (0.3) |  | 4.6 | (0.2) |  | *2.2* | (0.2) |

**Legend S2 Table.**  Percentage values show represent the number of adults meeting each definition all combined aerobic and strengthening activity used. SE standard error for the percentage values shown as an estimate of the England adult population aged 19-65 years. HSE - health survey for England evidence represents 150 minutes of moderate activity or equivalent plus twice weekly base of strength and then activity for which there was an evidence of health benefit guideline is purposeful strengthening exercises as defined in the current UK physical activity guidelines IMD indices of multiple deprivation derive from post code to provide area level scores at local board level or lower super output group Q1 represents the least deprived open brackets more affluent close brackets with five representing the most deprived areas highest educational qualification achieved level 1 basic education level 2 secondary education Level 3 further education and level 4 higher education studying two bachelors level; disability status relates to physical disability and was self-reported and classified as noticeability or able bodied a non-limiting disability inc and a limiting disability if the condition was reported to have a significant impact on tasks of daily living.
