# Supplementary material for: Who is meeting the strengthening physical activity guidelines by definition: A cross-sectional study of 253 423 English adults?
Source: PLoS One. 2022 May 4;17(5):e0267277. doi: 10.1371/journal.pone.0267277 (PMC9067886; doi:10.1371/journal.pone.0267277)
Supplement: S4 Table — (DOCX) [file pone.0267277.s004.docx]

**S4 Table Comparative sensitivity analysis for predictors of aerobic and strengthening activity including aerobic activity values greater than 2280 minutes per week**

|  | Aerobic Activity | | | | Strengthening Activity | | | Aerobic & Strengthening | | |
| --- | --- | --- | --- | --- | --- | --- | --- | --- | --- | --- |
| Sex | PR | (95%CI) | | | PR | (95%CI) | | PR | (95%CI) | |
| Male | 1.00 |  | | Ref | 1.00 |  | Ref | 1.00 |  | Ref |
| Female | 0.87 | (0.86 | | 0.88) | 0.67 | (0.66 | 0.68) | 0.75 | (0.73 | 0.77) |
| Age |  |  | |  |  |  |  |  |  |  |
| 19-34 | 1.00 |  | Ref | | 1.00 |  | Ref | 1.00 |  | Ref |
| 35-49 | 0.89 | (0.89 | | 1.01) | 0.77 | (0.74 | 0.80) | 0.98 | (0.97 | 0.99) |
| 50-64 | 0.98 | (0.97 | | 1.00) | 0.54 | (0.51 | 0.57) | 0.94 | (0.93 | 0.97) |
| Deprivation |  |  | |  |  |  |  |  |  |  |
| Least Deprived | 1.00 |  | Ref | | 1.00 |  | Ref | 1.00 |  | Ref |
| 2^nd^ Least Deprived | 0.96 | (0.93 | | 0.98) | 0.92 | (0.90 | 1.00) | 0.98 | (0.96 | 0.99) |
| Median Quintile | 0.93 | (0.91 | | 0.97) | 0.91 | (0.89 | 0.96) | 0.95 | (0.93 | 0.97) |
| 2^nd^ Most Deprived | 0.89 | (0.86 | | 0.92) | 0.87 | (0.80 | 0.91) | 0.93 | (0.91 | 0.90) |
| Most Deprived | 0.86 | (0.85 | | 0.89) | 0.81 | (0.79 | 0.88) | 0.83 | (0.80 | 0.90) |
| Education |  |  | |  |  |  |  |  |  |  |
| ≤ Level 1 | 1.00 | Ref | | | 1.00 |  | Ref | 1.00 |  |  |
| = Level 2 | 1.19 | (1.17 | | 1.21) | 1.28 | (1.23 | 1.29) | 1.18 | (1.15 | 1.20) |
| = Level 3 | 1.28 | (1.24 | | 1.30) | 1.57 | (1.45 | 1.70) | 1.28 | (1.25 | 1.30) |
| ≥ Level 4 | 1.36 | (1.33 | | 1.38) | 1.61 | (1.49 | 1.73) | 1.36 | (1.33 | 1.38) |
| Disability |  |  | |  |  |  |  |  |  |  |
| No Disability | 1.00 |  | | Ref | 1.00 |  | Ref | 1.00 |  | Ref |
| Non-Limiting | 1.01 | (0.99 | | 1.08) | 1.01 | (0.98 | 1.05) | 1.02 | (0.98 | 1.06) |
| Limiting disability | 0.83 | (0.68 | | 0.95) | 0.66 | (0.58 | 0.69) | 0.80 | (0.59 | 0.98) |

**S4 Table Legend**

a-Quintiles based on the Index of Multiple Deprivations (IMD) derived from postcode to provide area level scores at local ward level (lower super output area) grouping. Q1 represents the least deprived (more affluent) with five representing the most deprived areas highest Educational qualification achieved: Level 1 basic education Level 2 Completed secondary education ; Level 3- Completed further education. Level 4 attended higher education studying to Bachelors level. Disability status relates to physical disability and was self-reported and classified as noticeability or able bodied a non-limiting disability inc and a limiting disability if the condition was reported to have a significant impact on tasks of daily living. 150 min/week MPA or equivalent (75 min) vigorous activity of any mix of the two -classification based on compositive physical activity measures MEMS7_ALL and MEMS7_ALL(GR) taken from the Active Lives Survey. (33)
