# Supplementary material for: Who is meeting the strengthening physical activity guidelines by definition: A cross-sectional study of 253 423 English adults?
Source: PLoS One. 2022 May 4;17(5):e0267277. doi: 10.1371/journal.pone.0267277 (PMC9067886; doi:10.1371/journal.pone.0267277)
Supplement: Attachment — Submitted filename: Responses to Reviewers PONE-D-21-24467 R1.docx [file pone.0267277.s005.docx]

PONE-D-21-24467

Who is meeting the strengthening physical activity guidelines by definition: a cross sectional study of 253423 English adults

PLOS ONE

Dear Dr. Sandercock,

Thank you for submitting your manuscript to PLOS ONE. After careful consideration, we feel that it has merit but does not fully meet PLOS ONE’s publication criteria as it currently stands. Therefore, we invite you to submit a revised version of the manuscript that addresses the points raised during the review process.

Please submit your revised manuscript by Feb 04 2022 11:59PM. If you will need more time than this to complete your revisions, please reply to this message or contact the journal office at [plosone@plos.org](mailto:plosone@plos.org). When you're ready to submit your revision, log on to https://www.editorialmanager.com/pone/ and select the 'Submissions Needing Revision' folder to locate your manuscript file.

Please include the following items when submitting your revised manuscript:

- A rebuttal letter that responds to each point raised by the academic editor and reviewer(s). You should upload this letter as a separate file labeled 'Response to Reviewers'.
- A marked-up copy of your manuscript that highlights changes made to the original version. You should upload this as a separate file labeled 'Revised Manuscript with Track Changes'.
- An unmarked version of your revised paper without tracked changes. You should upload this as a separate file labeled 'Manuscript'.

If you would like to make changes to your financial disclosure, please include your updated statement in your cover letter. Guidelines for resubmitting your figure files are available below the reviewer comments at the end of this letter.

If applicable, we recommend that you deposit your laboratory protocols in protocols.io to enhance the reproducibility of your results. Protocols.io assigns your protocol its own identifier (DOI) so that it can be cited independently in the future. For instructions see: <https://journals.plos.org/plosone/s/submission-guidelines#loc-laboratory-protocols>. Additionally, PLOS ONE offers an option for publishing peer-reviewed Lab Protocol articles, which describe protocols hosted on protocols.io. Read more information on sharing protocols at <https://plos.org/protocols?utm_medium=editorial-email&utm_source=authorletters&utm_campaign=protocols>.

We look forward to receiving your revised manuscript.

Kind regards,

Martin Senechal, PhD
Academic Editor
PLOS ONE

Journal requirements:

1. When submitting your revision, we need you to address these additional requirements.

Please ensure that your manuscript meets PLOS ONE's style requirements, including those for file naming. The PLOS ONE style templates can be found at

https://journals.plos.org/plosone/s/file?id=wjVg/PLOSOne_formatting_sample_main_body.pdf and

https://journals.plos.org/plosone/s/file?id=ba62/PLOSOne_formatting_sample_title_authors_affiliations.pdf

2. Please note that to use the direct billing option the corresponding author must be affiliated with the chosen institute. Please either amend your manuscript to change the affiliation or corresponding author or email us at plosone@plos.org with a request to remove this option.

This is now the case

3.  In your Data Availability statement, you have not specified where the minimal data set underlying the results described in your manuscript can be found. PLOS defines a study's minimal data set as the underlying data used to reach the conclusions drawn in the manuscript and any additional data required to replicate the reported study findings in their entirety. All PLOS journals require that the minimal data set be made fully available. For more information about our data policy, please see http://journals.plos.org/plosone/s/data-availability.

These details are now included in our cover letter

Upon re-submitting your revised manuscript, please upload your study’s minimal underlying data set as either Supporting Information files or to a stable, public repository and include the relevant URLs, DOIs, or accession numbers within your revised cover letter. For a list of acceptable repositories, please see http://journals.plos.org/plosone/s/data-availability#loc-recommended-repositories. Any potentially identifying patient information must be fully anonymized.

We have taken the second of these options – we do not have a license to share Sport England’s data *per se* but we have provided stable URLs and DOIs to enable researchers to access the full raw data via the UK data repository.

Important: If there are ethical or legal restrictions to sharing your data publicly, please explain these restrictions in detail. Please see our guidelines for more information on what we consider unacceptable restrictions to publicly sharing data: http://journals.plos.org/plosone/s/data-availability#loc-unacceptable-data-access-restrictions. Note that it is not acceptable for the authors to be the sole named individuals responsible for ensuring data access. We will update your Data Availability statement to reflect the information you provide in your cover letter.

Reviewers' comments:

Reviewer's Responses to Questions

**Comments to the Author**

1. Is the manuscript technically sound, and do the data support the conclusions?

The manuscript must describe a technically sound piece of scientific research with data that supports the conclusions. Experiments must have been conducted rigorously, with appropriate controls, replication, and sample sizes. The conclusions must be drawn appropriately based on the data presented.

Reviewer #1: Yes

2. Has the statistical analysis been performed appropriately and rigorously?

Reviewer #1: Yes

3. Have the authors made all data underlying the findings in their manuscript fully available?

The [PLOS Data policy](http://www.plosone.org/static/policies.action#sharing) requires authors to make all data underlying the findings described in their manuscript fully available without restriction, with rare exception (please refer to the Data Availability Statement in the manuscript PDF file). The data should be provided as part of the manuscript or its supporting information, or deposited to a public repository. For example, in addition to summary statistics, the data points behind means, medians and variance measures should be available. If there are restrictions on publicly sharing data—e.g. participant privacy or use of data from a third party—those must be specified.

Reviewer #1: Yes

4. Is the manuscript presented in an intelligible fashion and written in standard English?

PLOS ONE does not copyedit accepted manuscripts, so the language in submitted articles must be clear, correct, and unambiguous. Any typographical or grammatical errors should be corrected at revision, so please note any specific errors here.

Reviewer #1: Yes

5. Review Comments to the Author

Please use the space provided to explain your answers to the questions above. You may also include additional comments for the author, including concerns about dual publication, research ethics, or publication ethics. (Please upload your review as an attachment if it exceeds 20,000 characters)

In the markup version of our revised manuscript, you will find responses to each of the comments below. wear these comments relate to minor changes to the wording they are shown in the document as track changes. in the case of additions or where it was necessary to undertake wholesale rewording of sections or paragraphs changes are shown in red rather than track changes to ensure readability.

The same system in our responses hello where responses to comments, where explanation is required, are shown in red and minor modifications based on your helpful comments as track changes.

Reviewer #1: Reviewer comments
This study aimed to provide estimates for strengthening activity prevalence in English adults and to quantify the variation in estimates attributable to differences in the way strengthening activity is defined. The study also aimed to study the association between sociodemographic factors and the performance of strengthening activities. The study is well written and addresses an important topic, i.e. how differences in prevalence estimates can be due to differences in the definitions of a behavior or condition. The statistical methods used are appropriate for the research question.

General comments:
1. In what way are the participants contacted about participation in the Active Lives Survey (ALS)?

We have included a section in the methods explaining that this is a push-to-web survey whereby respondents Receive three invitations to take part in the study two of which asked him to fill it in online the third of which asked him to fill it in online again and then provides them with a hard copy of the study which they can return by free post.

2. What proportion of persons who are asked to participate in the ALS decline participation? Is there any data on the non-participants of the ALS, for example regarding age and sex distribution? In what way could the reward of a £5 shopping voucher affect which groups of persons choose to participate in the ALS?

We have included the response rate (19%)

As per the effect of the £5 voucher, this answer we have unavoidably avoided as we do not know the answer. we have however included information about how the survey is sent out in waves to ensure that it is representative of the English population in the limitations section. we hope that this together with the added detail that we have provided in the methods about how the survey is administered is satisfactory.

3. There is no reference to the supporting information in the text. Please add a sentence in the methods section indicating that additional information on the activities included in each definition of strengthening activities can be found in the supporting information. Instead, on line 211, there is a reference to Table 1 which does not include the definitions of strengthening activities.

I think apologies are in order here this should have been table S1 and we have now made reference to all supporting material including this table this one table S2 and the new table S3 which we have included, based on your suggestion to perform a sensitivity analysis. For simplicity and brevity, this takes the form of a simple repeating of the original analysis with the same reporting standards. Table S3, therefore, contains the same exponential parameters estimates as table 2 with the previously excluded cases included in the analysis.

REFERENCE to Table S1 IS NOW MADE IN LINE 222

REFERENCE to Table S2 IS Now made in LINE 250

We have now included Table S3-as per your suggestions of a sensitivity analysis (see responses below).

REFERENCE to Table S3 is now made in lin 618

4. I suggest that you consider performing a sensitivity analysis of the association between sociodemographic factors and meeting strengthening guidelines where participants reporting very high levels of aerobic physical activity are included. Would the inclusion of these individuals, who probably have a high level of physical activity, although most likely not as high as reported, change your results? Are the excluded individuals similar to the included individuals in characteristics such as age and sex?

We have taken up your suggestion to perform a sensitivity analysis. For simplicity and brevity this takes the form of repeating the original analysis, we have used the same reporting standards and included the results in the Supporting Information (Table S3) which contains the same exponential parameter estimates as table 2 with the previously excluded cases included in the analysis.

5. Please check that all abbreviations are spelled out at first use. Now Checked and shown as track changes in mark up.

Comments on specific sections of the manuscript:
Line 28: Please change ”quantifying” to ”quantify” changed to ‘quantify’
Line 29: The word “in” is missing changed to: ‘differences ‘in’ the way’
Line 42: The word ”women” is missing ‘of women’ now added
Lines 277-278: Please replace “open brackets”, “close brackets” with actual brackets. Apologies for this – it seems some coding from MacBook to Windows has taken ‘brackets’ as a word? Now changed.
Line 353: Please replace “open brackets”, “close brackets” with actual brackets. Again Apologies for this – it seems some coding from MacBook to Windows has taken ‘brackets’ as a word? Now changed.
Lines 365-368 and 373: The meaning of ”Strengthening guidelines” changed to ‘meeting strengthening acvtivity guidelines’ to fit with the rest of the text and “Aerobic and combined guidelines” is not immediately apparent. Please consider explaining this more clearly.
Line 370: Please change “slightly” to “likely”. Changed slightly to ‘likely’
Line 457: Please change “thae” to “that” Changed from ‘that thae’ to ‘that the’
Lines 538-539: “more pronounced associations between strengthening activity than for either aerobic activity (or combined aerobic and strengthening activity).” The meaning of this statement is unclear. Do you mean the association between the sociodemographic measures and level of strengthening activity? Yes, that’s what we meant – now changed Please consider rephrasing this statement.
Lines 539-540: “Differences in strengthening activity were much more pronounced in middle-aged adults and women”. Please elaborate. What was the direction of the differences mentioned and what was the comparison group? As above – this section has now been changed to provide a more expansive explanation of the direction of association observed including details of the referent groups in each category.
Line 550: Please change “at” to “as” now changed
Lines 550-553: If possible, please provide references supporting these theories. If there are no previous studies of the accessibility/perceived accessibility of gyms and resistance training facilities for women, older persons, and adults with disabilities, please state this. There are a number of studies in this area investigating barriers to participation in different modes of sports and exercise – we have now included three references in relation to disability, gender/age and socio-ecomonimic status.
Line 615: “national prevalence estimates”. Please state what prevalence was estimated.Wording changed

While revising your submission, please upload your figure files to the Preflight Analysis and Conversion Engine (PACE) digital diagnostic tool, <https://pacev2.apexcovantage.com/>. PACE helps ensure that figures meet PLOS requirements. To use PACE, you must first register as a user. Registration is free. Then, login and navigate to the UPLOAD tab, where you will find detailed instructions on how to use the tool. If you encounter any issues or have any questions when using PACE, please email PLOS at [figures@plos.org](mailto:figures@plos.org). Please note that Supporting Information files do not need this step.

*In compliance with data protection regulations, you may request that we remove your personal registration details at any time.* [*(Remove my information/details)*](https://www.editorialmanager.com/pone/login.asp?a=r)*. Please contact the publication office if you have any questions.*
